# Unravelling the temporal and spatial variation of fungal phylotypes from embryo to adult stages in Atlantic salmon

**DOI:** 10.1038/s41598-023-50883-x

**Published:** 2024-01-10

**Authors:** Jep Lokesh, Prabhugouda Siriyappagouder, Jorge M. O. Fernandes

**Affiliations:** 1 Faculty of Biosciences and Aquaculture, Nord University, Bodø, Norway; 2 Present Address: Université de Pau et des Pays de l’Adour, E2S UPPA. INRAE, NUMEA, Saint-Pée-Sur-Nivelle, France

**Keywords:** Applied microbiology, Microbiology, Microbial communities, Microbiome, Symbiosis

## Abstract

Early microbial colonization has a profound impact on host physiology during different stages of ontogeny. Although several studies have focused on early bacterial colonization and succession, the composition and role of fungal communities are poorly known in fish. Here, we sequenced the internal transcribed spacer 2 (ITS2) region of fungi to profile the mycobiome associated with the eggs, hatchlings and intestine of Atlantic salmon at various freshwater and marine stages. In most of the stages studied, fungal diversity was lower than bacterial diversity. There were several stage-specific fungal phylotypes belonging to different stages of ontogeny but some groups, such as *Candida tropicalis*, *Saccharomyces cerevisiae*, *Alternaria metachromatica*, *Davidiella tassiana* and *Humicola nigrescens*, persisted during successive stages of ontogeny. We observed significant changes in the intestinal fungal communities during the first feeding. Prior to first feeding, *Humicola nigrescens* dominated, but *Saccharomyces cerevisiae* (10 weeks post hatch) and *Candida tropicalis* (12 weeks post hatch) became dominant subsequently. Seawater transfer resulted in a decrease in alpha diversity and an increase in *Candida tropicalis* abundance. We also observed notable variations in beta diversity and composition between the different farms. Overall, the present study sheds light on the fungal communities of Atlantic salmon from early ontogeny to adulthood. These novel findings will also be useful in future studies investigating host-microbiota interactions in the context of developing better nutritional and health management strategies for Atlantic salmon farming.

## Introduction

The host-associated microbiome occupies a diverse ecological niche on various mucosal and non-mucosal body surfaces of different organisms. Microbial communities on mucosal surfaces, especially the intestinal microbiome, are involved in various biological processes, such as nutrient uptake, metabolism, energy homeostasis and regulation of the host immune system^1^. The intestinal microbiome is characterised by a remarkable diversity that includes a wide range of microorganisms from the kingdoms of Monera, Fungi and Protista as well as viruses. This diversity and the composition of the intestinal microbiome are host-specific, and generally (eu)bacteria and fungi in particular contribute significantly to the overall gene content within the community^2^. Although previous studies on the intestinal microbiome have mainly focused on the description and functional characterisation of bacterial communities, there is growing interest in the composition and functions of fungal groups (mycobiome) and their involvement in nutrition, health and disease in mammals^3,4^.

Fungi are eukaryotic microorganisms that generally have larger (compared to bacteria) and complex genomes, and are known to interact with the host immune system via specific receptors^5^. In human, a dysbiotic mycobiome has been associated with the development of irritable bowel syndrome and psychiatric disorders such as schizophrenia^3^. Functional studies have shown the specific enzyme repertoire of fungi involved in the digestion of resistant fibres in the rumen microbiome of livestock^4^. Furthermore, functional complementarity between fungal enzymes and specialized plant cell wall-degrading enzymes of rumen bacteria has also been demonstrated^4^.

The intestinal fungal population show high degree of variation compared to the bacterial microbiome^6^. The most important factors influencing the composition of the intestinal mycobiome include diet and ontogeny stage (age)^5^. For example, in humans, a plant-based diet causes an increase in the relative abundance of *Candida*, while consumption of a diet rich in animal foods leads to an increase in *Penicillium* species^7^. A similar effect of diet on fungal populations has been demonstrated in dairy cows, where the transition from a diet high in forage to a diet rich in cereals affected the abundance of the genera *Cyllamyces* and *Caecomyces*^8^. Regarding the effects of ageing on the mycobiome, *Malassezia*, *Saccharomyces* and *Debaryomyces hansenii* were the more common fungal genera in intestine of neonates aged 1–4 months, while *Saccharomyces* and *Candida* were the dominant fungal species in adults^9^. Moreover, such an age-related transition in the rumen mycobiome of dairy cows has also been reported^8^. On the other hand, there was no significant effect of age on the diversity of fungal communities in *Panthera tigris*^10^, suggesting that the age-related transition in the mycobiome might be species- and developmental stage-specific. A direct comparison of the composition and abundance profile of the mycobiome between mammals and teleosts is challenging due to differences in their living environments, dietary habits, anatomy, physiology of the digestive tract, immune systems, etc. Nevertheless, we hypothesize that ontogenic transition could strongly influence the fungal composition in teleosts, as observed in mammals^8,9^.

To date, there are very few studies on the mycobiome of teleosts, particularly in commercially important aquatic species. Knowledge of commensal fungal communities and the effects of factors such as age, diet and environmental conditions on community stability and functions is most useful from both basic and applied perspectives. A study in the teleost vertebrate model zebrafish showed that the intestinal tract comprises diverse mycobiota whose composition varies with habitat^11^. Furthermore, exposure of early developmental stages of zebrafish to commensal gut fungi had a significant impact on bacterial populations and expression of genes involved in metabolism and immunity^12,13^. Studies on the intestinal fungal communities of tilapia (*Oreochromis mossambicus*), an omnivorous species, and bighead carp (*Aristichthys nobilis*), a filter-feeding herbivorous species, have shown that the fungal groups are host-specific, with a significant difference in beta diversity, possibly driven by the feeding habits of the fish^14^. The symbiosis between the fungal groups and the fish host is also implicated in the wood-eating fish *Panaque nigrolineatus*^15^, whereas the diet (frozen fish vs. formulated feed) did not affect the intestinal fungal populations of cobia fish (*Rachycentron canadum*)^16^. Furthermore, a core group of fungi, including *Debaryomyces hansenii* and *Rhodotorula mucilaginosa*, has been identified in both wild and cultivated carnivorous fish species, such as Atlantic salmon (*Salmo salar*), rainbow trout (*Oncorhynchus mykiss*), Coho salmon (*Oncorhynchus kisutch*), corvina drum (*Cilus gilberti*), and cape yellowtail (*Seriola lalandi*)^17^.

Atlantic salmon is one of the most important species in aquaculture. Its life cycle include both freshwater and seawater phases, providing a unique opportunity to study the progression of the mycobiome with age and various physiological transitions^18^. We have previously demonstrated the transition of bacterial community composition in the early embryonic stages and intestine of Atlantic salmon in both freshwater and seawater phases^18^. We hypothesize that there will be a similar transition in the fungal community. Hence in the present study, we investigated the early colonization and succession of fungal communities associated with ontogeny in embryonic stages, hatched larvae and intestine of Atlantic salmon at various freshwater and seawater stages by amplicon sequencing of the fungal internal transcribed spacer 2 (ITS2) region using the Illumina MiSeq platform. To understand spatial variation in adult Atlantic salmon fungal communities, samples were also collected from rearing farms at different locations.

## Materials and methods

### Sampling

#### Ethical approval

This study was conducted in accordance with the guidelines of the Norwegian Animal Research Authority**.** The animal experimentation protocols used in the study were approved by Norwegian Animal Research Authority (FDU; approval number: 7899)**.** All methods are reported in accordance with ARRIVE guidelines.

#### Ontogeny stages

Samples were collected from different freshwater and seawater stages of ontogeny from hatchery-reared Atlantic salmon (Cermaq AS, Bodø, Norway). All samples were collected from a single cohort of Atlantic salmon. All metadata on the samples and the stages selected for sampling are presented in Lokesh et al.^18^. The salinity, temperature and pH of the hatchery rearing water were around < 0.5 ppt, 12 °C and 7, respectively, whereas the conditions in the seawater rearing system were 34 ppt, 12–13 °C and 7, respectively.

A commercial starter feed (Biomar, Aarhus, Denmark) was provided to the first feeding fry. The pellet size was adjusted as the fish grew. Fish undergoing smoltification were fed smolt feed (Biomar). After the transfer to seawater, the fish were given grower's feed (Biomar). In commercial farms, fish were fed with grower feed from Skretting, Stavanger, Norway. These diets are standardized to meet the nutritional needs of the fish at various stages of ontogeny, ensuring appropriate levels of macro and micronutrients^19^. The nutrient compositions are given in the Supplementary Table S1. The samples of embryos and hatchlings (whole) in the present study came from trays housed in a single tank. The fish (for intestine sampling) from the other ontogeny stages were taken from a single tank in which they were being reared. All samples (n = 10 from each ontogeny stage) were collected after either the embryos/fish had been euthanised with 200 mg/L MS-222 (tricaine methanesulfonate; Argent Chemical Laboratories, Redmond, USA). Samples of the eyed eggs [EE; 240 degree-days (DD)], eggs one week before hatching (EBH; 396 DD) and hatchlings (HL; 480 DD) were taken at the early stages of development. At 7 weeks post hatch (wph) when yolk sac absorption was complete, the entire intestine was visible and sampled until 12 wph to understand the transition of fungal communities in the early freshwater stages. First the fish were euthanised and then the fish were placed under a magnifying glass. The belly was cut open and the intestine was carefully taken out using sterile tools. The harvested intestine was placed in a cryotube and flash frozen in liquid nitrogen. The distal intestine was clearly distinguishable from 20 wph and was sampled from this point onwards for both freshwater stages (20, 44 and 62 wph) and marine stages (65, 68 and 80 wph). The distal intestine was cut open using sterile tools, and both its contents and mucus were collected. All samples were transferred to a cryotube, immediately frozen in liquid nitrogen and stored at − 80 °C until DNA extraction. A graphical representation of the difference stages sampled is given in Lokesh et al.^18^.

#### Farms

To understand spatial variation in the mycobiome, adult fish were collected from three different farms in the Nordland region, Norway. Of the three farms, two (farms 1 and 2) were located in Hamarøy (67.8954° N, 15.9416° E) and farm 3 was located in Inndyr (67.0319° N, 14.0281° E). The average weight of sampled fish was 3.60 ± 0.41 kg, 3.79 ± 0.58 kg, and 2.83 ± 0.63 kg for farms 1, 2 and 3, respectively. Fish were sampled (n = 10 from each farm) after being euthanized with a lethal dose (200 mg/L) of MS-222. The intestine was aseptically dissected, and its contents and mucus were collected by scraping with a sterile glass slide. Samples were snap-frozen in liquid nitrogen and stored at − 80 °C until DNA extraction.

### DNA extraction, preparation and sequencing of libraries

DNA from the samples was extracted using the QIAamp DNA Stool Mini Kit (Qiagen, Nydalen, Sweden) according to the manufacturer's protocol and modifications by Lokesh and Kiron^20^. Amplicon libraries were prepared according to the dual-index protocol described by Kozich et al.^21^ using the fungus-specific primers fITS7: GTGARTCATCGAATCTTTG and ITS4: TCCTCCGCTTATTGATATGC^22^. PCR was performed using a T100 PCR thermal cycler (Bio-Rad, Oslo, Norway) in a 25 μL reaction volume containing 12.5 μL Kapa HiFi HotStart PCR Ready Mix (KAPA biosystems, Woburn, USA), 2.5 μL forward and reverse primers (300 nM) and 7.5 μL of the DNA elutions. Thermocycling conditions included an initial denaturation step at 95 °C for 5 min, followed by 30 cycles at 98 °C for 30 s, annealing at 58 °C for 30 s and extension at 72 °C for 45 s. A final extension was performed at 72 °C for 2 min. PCR reactions were performed in triplicate and included negative and positive controls. The positive controls contained ZymoBIOMICS Microbial Community DNA Standard (Zymo Research, Irvine, USA), while the negative controls contained water in place of samples. After completion of PCR, triplicate reactions per sample were pooled and further diluted and quantified prior to sequencing^18,21^. Amplicon libraries were sequenced at a concentration of 10 pM with an equimolar 10% PhiX control library on a MiSeq platform (Illumina Inc., San Diego, CA, USA) using the MiSeq v3 reagent kit (300 bp paired-end). The 16S rRNA data used for comparing bacterial and fungal diversities were generated as part of a separate study^18^. Briefly, the same dual-index method used to generate fungal ITS amplicons was applied to generate 16S rRNA amplicons using the primers V3-341F-CCTACGGGAGGCAGCAG and V4-785R-GGACTACHVGGGTWTCTAAT to amplify the V3–V4 region of the 16S rRNA gene. The resulting data were analyzed using the UPARSE pipeline to obtain the OTUs^23^.

### Sequence and statistical analysis

The resulting sequence data were used for downstream analysis. The forward and reverse reads were trimmed to 260 and 270 base pairs, respectively. These lengths were selected based on the average Phred scores, which tended to be lower than Q30 after 260 and 270 bp for the forward and reverse reads, respectively. The trimmed reads were merged and quality filtered using expected error filtering strategy (maximum expected error rate threshold = 0.01) described in the UPARSE pipeline^23^ and further processed with the PIPITS pipeline using default parameters^24^. OTUs with a similarity of 97% were created, which in turn were assigned taxonomy using the RDP classifier^25^ and the UNITE database^26^. The phylotype table created with these data was used for analysis of underlying diversity and community structure. Count data were divided into five categories based on the type and origins of sample, namely early developmental stages (EE, EBH and HL), whole intestine from freshwater stages (7 wph, 8 wph, 10 wph and 12 wph), distal intestine from freshwater stages (20 wph, 44 wph and 62 wph), distal intestine from seawater stages (65 wph, 68 wph and 80 wph), and the farms (farm 1, farm 2 and farm 3). All downstream analyses were performed separately for these five categories. Alpha diversity and beta diversity measures were computed using the R^27^ package Phyloseq, version 1.12.2^28^. Statistical significance of differences in alpha diversity indices is evaluated using R. When data were normally distributed with equivariance, the parametric one-way ANOVA was used. If the assumptions of either normal distribution or equal variance were not met, a non-parametric one-way ANOVA (Kruskal–Wallis test) was used. Tukey's HSD was used to perform the post-hoc test. Beta diversity was calculated using Bray–Curtis dissimilarity index. Statistical significance of dissimilarity was assessed using ANOSIM: analysis of similarity^29^. Prior to ANOSIM, the significance of differences in dispersion between groups was assessed using the betadisper function in the R package vegan, version 2.5.1^30^.

Core members of the mycobiome were computed using QIIME^31^ by identifying phylotypes present in at least 90% of samples belonging to a group (either a life stage or a farm) with abundance more than 0.01%. Significantly abundant core phylotypes belonging to a given group were identified using linear discriminant analysis effect size (LEfSe) with a p-value cut-off of 0.05 (for the paired t-test and Wilcoxon rank-sum test) and an LDA cut-off of 4^32^. Alpha diversity indices of bacterial OTUs were determined according to the protocol described in Lokesh et al.^18^.

## Results

### Overview of the sequence data

We sampled 10 individuals from their respective stages. Results for all 10 samples are presented for most stages, except for EE = 8, 80wph = 7, Farm1 = 8, and Farm3 = 9. Some samples were excluded from the final dataset due to extremely low sequence coverage. We obtained a total of 7,673,614 quality-filtered fungal ITS2 sequences from 150 samples, which were clustered into 915 OTUs. Further, we obtained 501 phylotypes from these OTUs by grouping the OTUs with the same taxonomy into a phylotype. Rarefaction depths were 7239, 21,386, 24,003, 21,062 and 11,632 for early developmental stages (EE, EBH and HL), whole intestine from freshwater stages (7 wph, 8 wph, 10 wph, 12 wph), distal intestine of fish from freshwater stages (20 wph, 44 wph, 62 wph), seawater stages (65 wph, 68 wph, 80 wph) and farms (farm 1, farm 2, farm 3), respectively (Supplementary Fig. S1A–E).

### The fungal diversity and composition of eggs, hatchlings, and the intestine during the early stages of ontogeny

**Figure 1:**
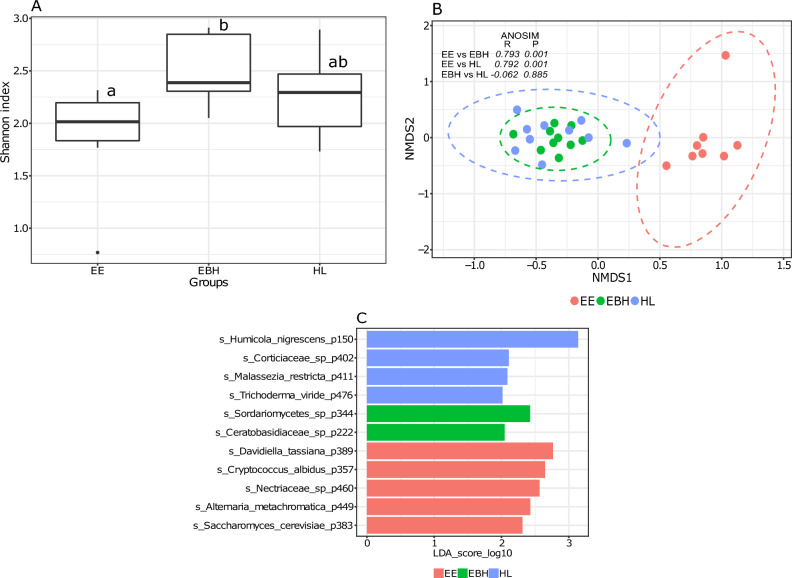
Boxplot showing fungal alpha diversity in terms of Shannon index during early developmental stages of Atlantic salmon, namely eyed egg (EE), eggs before hatching (EBH), and hatchlings (HL) (**A**). Different letters above the boxplot indicate significant differences between groups (P < 0.05). NMDS plot showing beta diversities calculated with the Bray–Curtis dissimilarity index (**B**). The differences in clustering patterns were analysed using analysis of similarities (ANOSIM), and *R* and *P* values for each of the comparisons are indicated. Significantly abundant core phylotypes (present in > 90% samples at > 0.01%) belonging to each of the ontogeny stages as identified by LEfSe (**C**).

Fertilized eggs at early developmental stages harbored a diverse fungal community, with pre-hatch eggs having a significantly higher Shannon diversity index (Fig. 1A, P < 0.05). Hatching had no significant effect on community richness and evenness, as indicated by the Shannon index (Fig. 1A, P > 0.05). The fungal beta diversity was significantly different between EE and EBH, and EE and HL groups in the early stages (Fig. 1B; *P* < 0.01, *R* > 0.7). Hatchlings did not differ significantly from the EBH in their beta diversity (*P* > 0.01). In the early developmental stages, there were a total of 33 core phylotypes in the three groups (Supplementary Fig. S2). Several of these core phylotypes were identified as significantly differentially abundant in different groups. The phylotypes *Davidiella tassiana* (0.25%), *Cryptococcus albidus* (0.14%), *Nectriaceae* sp. (0.14%), *Alternaria metachromatica* (0.1%) and *Saccharomyces cerevisiae* (0.085%) were most significant in EE, while *Sordariomycetes* sp. (0.09%) and *Ceratobasidiaceae* sp. (0.04%) were significantly abundant in EBH. *Humicola nigrescens* (0.48%), *Corticiaceae* sp. (0.03%), *Malassezia restricta* (0.05%), *Trichoderma viride* (0.03%) were found to be significant phylotypes in the HL group (Fig. 1C, Supplementary Fig. S2).

**Figure 2:**
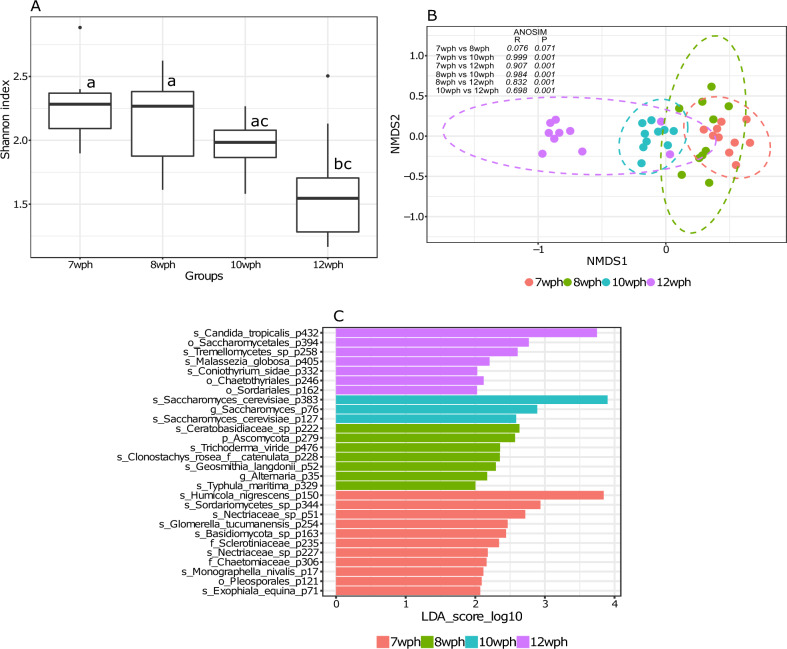
Boxplot showing fungal alpha diversity in the intestine of Atlantic salmon at early stages of ontogeny (7 wph, 8 wph, 10 wph, and 12 wph) (Shannon index) (**A**). Different letters above the boxplot indicate significant differences between groups (P < 0.05). NMDS plot showing beta diversities calculated with the Bray–Curtis dissimilarity index (**B**). The differences in clustering patterns were analysed using analysis of similarities (ANOSIM), and *R* and *P* values for each of the comparisons are indicated. Significantly abundant core features (present in > 90% samples at > 0.01%) belonging to each of the stages of ontogeny as identified by LEfSe (**C**).

Intestine samples collected at early ontogeny stages (7 wph and 8 wph) of Atlantic salmon showed relatively high species richness and evenness (Fig. 2A, P < 0.05), which decreased significantly following 4 weeks feeding (12 wph) (Fig. 2A, P < 0.05). There were no significant differences in beta diversity between samples collected at 7 wph and 8 wph (Fig. 2B, P > 0.05), while samples collected at 10 wph and 12 wph were significantly different from each other as well as the 7 wph and 8 wph showing a progressive age dependent transition. Overall, intestinal fungal communities of salmon differ from each other in the early freshwater stages (Fig. 2B, P < 0.01, *R* > 0.7). A total of 46 core phylotypes were identified in the intestine of Atlantic salmon at this phase (Supplementary Fig. S3). Of these 11, 7, 3 and 7 phylotypes were differentially abundant in samples collected at 7, 8, 10 and 12 wph, respectively. *Humicola nigrescens* (7 wph), *Saccharomyces cerevisiae* (10 wph), and *Candida tropicalis* (12 wph) were the most significant phylotypes depending on the specific early freshwater stages of Atlantic salmon (Fig. 2C).

### Distal intestinal fungal populations of Atlantic salmon in late freshwater stages

**Figure 3:**
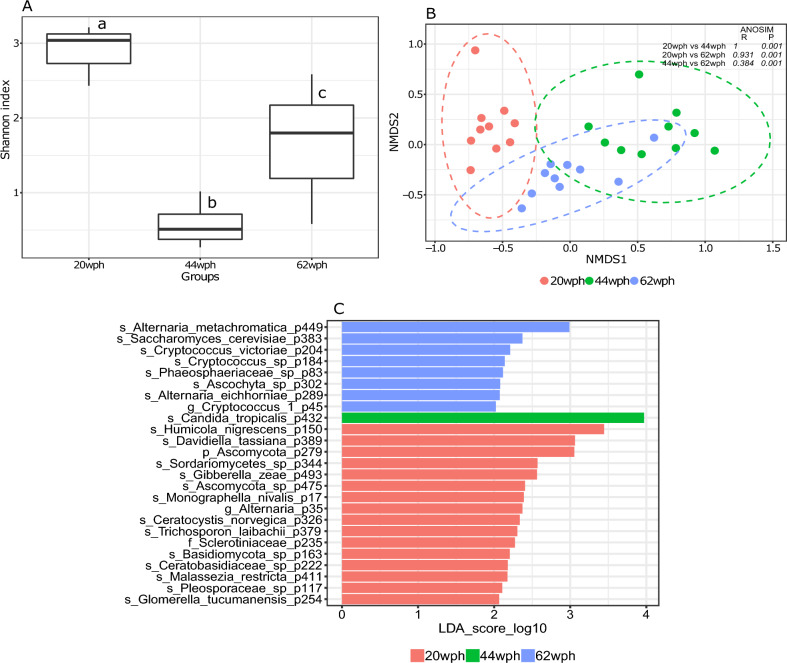
Boxplot showing fungal alpha diversity in the intestine of Atlantic salmon at late freshwater stages (20 wph, 44 wph, and 62 wph) (Shannon index) (**A**). Different letters above the boxplot indicate significant differences between groups (P < 0.05). NMDS plot showing beta diversities calculated with the Bray–Curtis dissimilarity index (**B**). The differences in clustering patterns were analysed using analysis of similarities (ANOSIM), and *R* and *P* values for each of the comparisons are indicated. Significantly abundant core features (present in > 90% samples at > 0.01%) belonging to each of the ontogeny stages as identified by LEfSe (**C**).

Species richness and evenness of the fungal communities were higher in the 20 wph, but decreased at 44 wph (Fig. 3A, P < 0.01). It increased towards the end of the freshwater stage at 62 wph but it was still significantly lower than the samples collected at 20 wph (Fig. 3A, P < 0.05). The beta diversity at 20 wph was significantly different from 44 and 62 wph (Fig. 3B, P < 0.01, *R* > 0.9). Samples collected at 44 wph and 62 wph were moderately similar to each other (*R* = 0.384). In total, 42 core phylotypes were identified among the three groups (Supplementary Fig. S4). Among the 25 significantly abundant core phylotypes, *Humicola nigrescens* (20 wph), *Davidiella tassiana* (20 wph), *Candida tropicalis* (44 wph), *Alternaria metachromatica* (62 wph) were the fungal phylotypes forming the most significant features in the distal intestine of Atlantic salmon at freshwater stages (Fig. 3C).

### Distal intestinal fungal populations of Atlantic salmon in seawater stages

**Figure 4:**
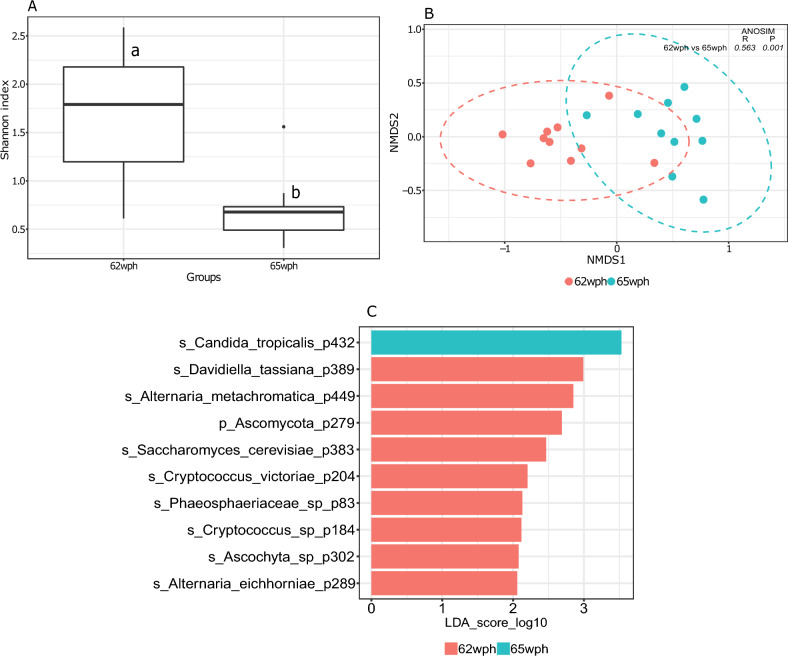
Boxplot showing fungal alpha diversity in terms of Shannon index during the transition between the freshwater and seawater stages of Atlantic salmon (62 wph and 65 wph) (**A**). Different letters above the boxplot indicate significant differences between groups (P < 0.05). NMDS plot showing beta diversities calculated with the Bray–Curtis dissimilarity index (**B**). The differences in clustering patterns were analysed using analysis of similarities (ANOSIM), and *R* and *P* values for each of the comparisons are indicated. Significantly abundant core features (present in > 90% samples at > 0.01%) belonging to each of the ontogeny stages as identified by LEfSe (C).

To understand the effect of seawater transfer, we compared the last stage sampled from freshwater with the first stage sampled in seawater. Samples collected from the late freshwater stage at 62 wph had higher fungal community richness and evenness than samples collected from the early seawater stage at 65 wph (Fig. 4A, P < 0.05). Clustering patterns of the intestinal fungal community of salmon during the freshwater (62 wph) and seawater (65 wph) phases were significantly different from each other (Fig. 4B, P < 0.01, *R* > 0.5). Out of the 22 core phylotypes identified in this phase (Supplementary Fig. S5), *Davidiella tassiana* (0.11%) and *Candida tropicalis* (0.88%) were the most significantly abundant phylotypes detected in the freshwater and seawater phases, respectively (Fig. 4C, Supplementary Fig. S5).

**Figure 5:**
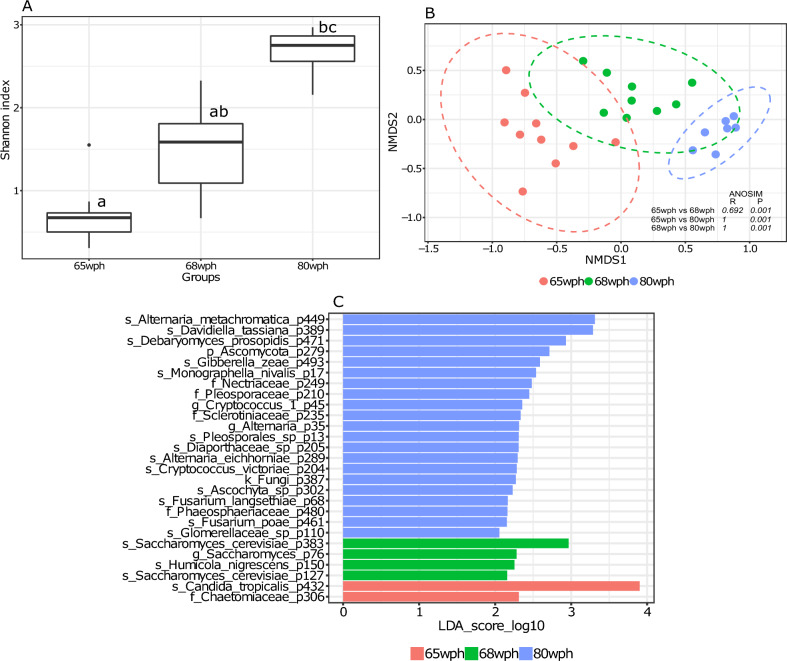
Boxplot showing fungal alpha diversity in the intestine of Atlantic salmon at seawater stages (65 wph, 68 wph, and 80 wph) (Shannon index) (**A**). Different letters above the boxplot indicate significant differences between groups (P < 0.05). NMDS plot showing beta diversities calculated with the Bray–Curtis dissimilarity index (**B**). The differences in clustering patterns were analysed using analysis of similarities (ANOSIM), and *R* and *P* values for each of the comparisons are indicated. Significantly abundant core features (present in > 90% samples at > 0.01%) belonging to each of the ontogeny stages as identified by LEfSe (**C**).

The Shannon diversity index of fungal communities in the distal intestine of Atlantic salmon was markedly different between seawater groups. The Shannon index was significantly higher in 80 wph compared to 65 wph (Fig. 5A, P < 0.05). Similarly, the beta diversities of the fungal communities in the intestine of seawater salmon were significantly different between stages (Fig. 5B, P < 0.05). *Alternaria metachromatica* (0.28%), *Saccharomyces cerevisiae* (0.1%), *Candida tropicalis* (0.89%) and *Davidiella tassiana* (0.2%) were the most significant fungal species among the 31 core phylotypes identified (Supplementary Fig. S6) in the intestine of salmon during seawater phases (Fig. 5C). It is noteworthy that after transfer to the seawater, the abundance of *Davidiella tassiana* (65 wph) comes down compared to 62 wph (Supplementary Fig. S5). Interestingly its abundance gradually increases in the late seawater stages and it becomes a significantly abundant phylotype in the 80 wph (Fig. 5C).

### Comparison of intestinal fungal communities in fish from different farms

**Figure 6:**
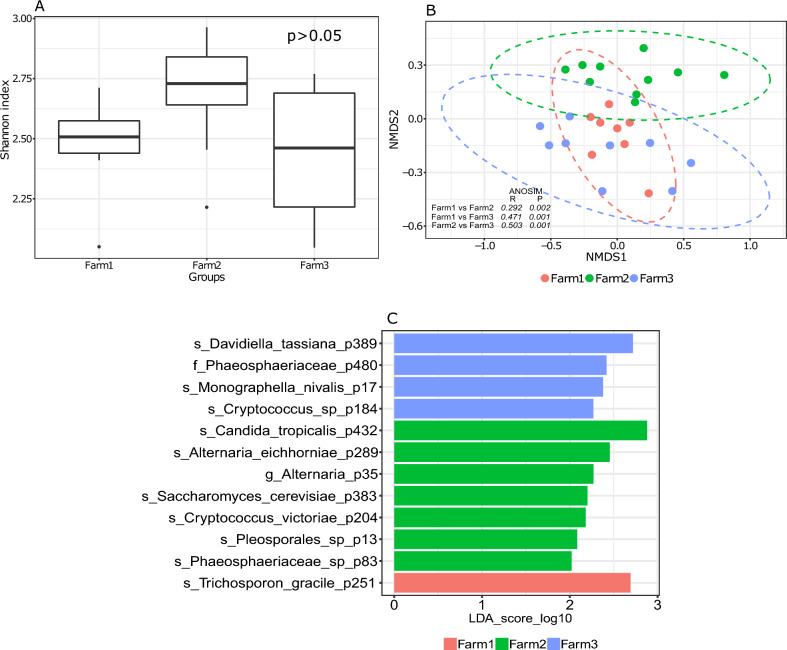
Boxplot showing fungal alpha diversity in the intestine of adult of Atlantic salmon in farms (farm 1, farm 2, and farm 3) (Shannon index) (**A**). Different letters above the boxplot indicate significant differences between groups (P < 0.05). NMDS plot showing beta diversities calculated with the Bray–Curtis dissimilarity index (**B**). The differences in clustering patterns were analysed using analysis of similarities (ANOSIM), and *R* and *P* values for each of the comparisons are indicated. Significantly abundant core features (present in > 90% samples at > 0.01%) belonging to each of the ontogeny stages as identified by LEfSe (**C**).

No significant differences in Shannon indices were observed for the intestinal fungal community of salmon from the three different farms (Fig. 6A). Beta diversity was moderately different among the three fish farms (Fig. 6B). There were 27 core fungal phylotypes, among which *Davidiella tassiana* and *Alternaria metachromatica* were abundant in all the farms (Supplementary Fig. S7). Twelve phylotypes were differentially abundant between different farms. *Trichosporon gracile* (found only in farm 1), *Candida tropicalis*, and *Davidiella tassiana* were the most significant members of farm 1, farm 2, and farm 3, respectively (Fig. 6C).

### Comparison between the bacterial and fungal diversities in Atlantic salmon intestine

Alpha diversity (Shannon index) of fungal OTUs was generally lower than the bacterial OTUs across different developmental stages, except EE, 8 wph, 20 wph, 62 wph and 68 wph. In the case of EBH and 80 wph, the fungal alpha diversity index was significantly higher than the bacterial diversity index. (Supplementary Fig. S8).

## Discussion

Here, we present, for the first time, a comprehensive analysis of the remarkable transitions occurring within fungal communities throughout the life cycle of Atlantic salmon. By examining different stages of ontogeny, we unravel the dynamic nature of fungal populations. Furthermore, we also assess the spatial variation of the mycobiome of Atlantic salmon across multiple farms.

### Hatching do not cause a change in the beta diversity of the mycobiome in Atlantic salmon

Salmonids have a long embryonic developmental time and the eggs are susceptible to fungal infections during this phase^33^. Therefore, there is immense interest in the commensal microbes present at these stages, as they could be used to combat pathogens. This potential of commensal organisms has already been demonstrated in Atlantic salmon, where a member of the Actinobacteria (genus: *Frondihabitans*) was able to limit the spread of *Saprolegnia* in eggs^33^. In the present study, EBH had the highest diversity (among EE, EBH and HL) and the diversity scores were higher than those of the bacterial population. This could indicate involvement of the fungal population in host developmental processes or pathogen containment^34^. On the contrary, the current study is the first to describe the fungal phylotypes associated with developing eggs using high-throughput sequencing technology. Through this sensitive method, it is possible to detect groups that were not previously reported. It is plausible that this diverse fungal community, even more extensive than bacteria, present in the eggs prior to hatching could be indicative of the presence of an array of opportunistic pathogenic groups^33^. Indeed, some members of the Malassezia, Sordariomycetes, Chaetomiaceae, and Ceratobasidiaceae, which were present in eggs, are known to encompass different plant and animal pathogens^35–38^. It is important to study the functional traits of salmon-specific species to understand the significance of their presence on the eggs. On the other hand, fish egg microbiota may influence embryonic development and hatching success^39,40^. A previous study noted significant changes in bacterial diversity during white fish egg development. Early stages exhibit high diversity similar to water communities, while diversity decreases later with increased host selection and shows a strong positive correlation with temperature^40,41^. In the present study, bacterial diversity remains stable pre-hatching, while fungal diversity peaks before hatching. Investigating molecular interactions between these fungal phylotypes and egg-associated microbiome could reveal their potential role in hatching success.

Interestingly, in contrast to the drastic changes in the bacterial population from EBH to HL^18^, there was no significant transition in the fungal composition from EBH to HL. This was surprising because hatching results in a complete change in the anatomical structures that normally supports independent populations^42^. Furthermore, after hatching, microenvironments such as the gills, mouth, and gut take shape and provide unique niches for specific phylotypes^43^. Although there were no changes in alpha- and beta diversity, the significant phylotypes belonging to HL were different, indicating a shift in the abundance of certain groups at the HL stage. This may indicate the involvement of these specific phylotypes in the development of the neural, digestive and immune system. Interactions between commensal bacteria and early ontogenic stages of zebrafish has been extensively studied and the specific bacterial species important for the fish development and survival are identified^44–47^.

Hatching is also a critical stage in ontogeny because the animal becomes more exposed to pathogenic organisms without protection from the chorion. Although members of *Malassezia restricta* found in the HL stage are not well characterized in teleosts, there are reports of this species causing infective endocarditis in humans^48^. On the other hand, *Trichoderma viride* (abundant in HL) is known to be a potent antifungal species that efficiently kills pathogenic fungi in plants through its extracellular secretions consisting mainly of the enzymes chitinases and β-1,3-glucanases^49^. It would be relevant to investigate whether the salmon phylotype has similar potential for pathogen control.

### First feeding has a strong impact on the intestinal fungal diversity and composition

The transition from yolk to exogenous feeding is a critical window in the ontogeny of teleosts including salmon^50^. In contrast to the non-significant transition in bacterial alpha and beta diversity, there were significant changes in the diversity of fungal populations. The difference in diversity (both alpha and beta) between the 7 wph and 8 wph was minimal. This could be due to the fact that the sampling points were only one week apart, which is not sufficient to detect inter-individual differences in the onset of feeding. The other comparisons between the stages sampled at this phase were significantly different from each other, indicating a progressive transition in the fungal communities. This suggests that the fungal populations are more sensitive to the onset of exogenous feeding than the bacterial populations. The progressive decrease in alpha diversity could be an indication of host selection of the phylotypes or the predominance of the phylotypes that could utilize the dietary components. Such selection by the host and the effect of diet is extensively reported for the bacterial microbiome in teleosts^43,51, 52^.

We sampled 3 late freshwater stages and observed a significant fluctuation in alpha diversities, with 44 wph showing a sharp decline in Shannon index. In addition, beta diversities also differed significantly between the three groups sampled in this phase, indicating a progressive transition in community composition with development. Although the factors that caused the sharp decline in alpha diversity are not known, it should be noted that there was one phylotype (*Candida tropicalis*) that was highly abundant at this stage. It needs to be further investigated whether such high abundance has negative effects on the occurrence of other groups. Although *Candida tropicalis* is known to interfere with biofilm formation and morphogenesis of *Candida albicans* in humans, its effect on other fungal groups is unknown^53^.

### Transfer of Atlantic salmon to seawater leads to a transition in fungal diversity and composition

Transfer to seawater is another critical step in the life history of Atlantic salmon, as the fish undergoes a series of physiological changes before entering seawater^54^. In the present study, a significant decrease in alpha diversity was observed after the fish were transferred to seawater. Beta diversity was also significantly different between the freshwater (62 wph) and seawater (65 wph) groups. It is known that the change to seawater affects the composition of the bacterial community both on the skin and intestine^20,55^. It is noteworthy that a clear shift in the phylogenetic content was observed in the case of the bacterial microbiome, whereas in the present study, the phylotypes observed as significant features of the seawater stages (80 wph) were consistently observed in other stages and their abundance increases in seawater, indicating the halophilic nature of the phylotypes.

Immediately after the seawater transfer, *Candida tropicalis* was identified as the most abundant phylotype in the seawater group (65 wph), although it was also present at a substantial level in the freshwater group (62 wph). This suggests that the *Candida tropicalis* strain found in Atlantic salmon thrives both in freshwater and seawater, with a preference for seawater as its abundance increased in seawater (65 wph). The three stages sampled during the seawater phase showed a gradual significant increase in alpha diversity indices. At the same time, beta diversities also differed significantly between the groups. The drastic reduction in alpha diversity immediately after transfer to seawater and the progressive increase in alpha diversity during the seawater phase could indicate a restructuring of the compositional profile in response to the changes in salinity^56,57^. Several phylotypes, including *Alternaria metachromatica*, *Davidiella tassiana* and *Debaryomyces prosopidis*, were identified as significant features of the late seawater stages (80 wph). It is noteworthy that these phylotypes were also present in fish at freshwater stages. It will be interesting to study the symbiotic relationships between these phylotypes and the host in different salinity levels to understand their importance in host biology.

### Spatial variations in the intestinal fungal communities of Atlantic salmon

The fungal communities from the three farms did not differ significantly from each other in terms of their alpha diversities, whereas their beta-diversities differed significantly from each other. The phylotypes observed in adult fish from the farms were broadly similar to those found in the early developmental stages and in the intestines of the juveniles, although the abundance of certain groups was significantly different between farms. Such differences in intestinal community composition between farms (spatial differences) could be due to several reasons, including water temperature, salinity, pH and other husbandry practices, as well as fish origin and host-derived factors^58,59^. Although there are not many studies showing the spatial variations in the intestinal fungal communities of teleosts, it has been shown that the fungal phylotypes in the wild and lab reared zebrafish are significantly different. The wild zebrafish being highly abundant with Dothideomycetes whereas the lab reared fish comprising mainly of Saccharomycetes^11^. In the present study, the adult Atlantic salmon exhibited a predominant presence of *Humicola nigrescens*, *Saccharomyces cerevisiae*, *Candida tropicalis*, *Davidiella tassiana*, and *Alternaria metachromatica*. In contrast, tilapia (*Oreochromis mossambicus*) and bighead carp (*Aristichthys nobilis*) from a single reservoir displayed the highest abundance of genera such as *Alternaria* (Dothideomycetes), *Scopulariopsis* (Sordariomycetes), and *Chaetomium* (Sordariomycetes)^14^. Cobia, on the other hand, showcased core dominant groups including *Debaryomyces*, *Saccharomyces*, *Ascobolus*, and *Cladosporium*^16^. These variations can be attributed to differences in intestinal anatomy and physiology, rearing temperature, salinity and feeding habits of the fish^60^. For instance, in wood-eating Amazonian catfish (*Panaque nigrolineatus*), the high abundance of *Fusarium oxysporum*, *Aureobasidium pullulans*, *Botrytis caroliniana*, *Metschnikowia*, *Alternaria*, and *Debaryomyces* has been linked to the fish's feeding behavior^15^. Notably, *Fusarium oxysporum*, which dominated the community, excretes endocellulases, exocellulases, and β-glucosidase, facilitating the degradation of cellulose^61^. Conversely, in cobia (*Rachycentron canadum*), different feed types (frozen fish vs. commercial extruded diet) had no discernible effect on the intestinal fungal population^16^. This finding suggests that the diet-dependent adaptation of fungal populations may be host-specific, highlighting the intricate relationship between the host and its fungal community.

### Dominant fungal communities of Atlantic salmon during their early and adult life

A few phylotypes were present in the fish irrespective of the stages of development or the samples source (different farms), namely *Humicola nigrescens*, *Saccharomyces cerevisiae, Candida tropicalis*, *Davidiella tassiana,* and *Alternaria metachromatica*. Their ubiquitous presence in all life stages, including the eggs and hatchlings, and the intestine in both freshwater and marine phases, suggests that they are subject to direct host selection and that these core members may be vital to host welfare^62^. Different strains of *Saccharomyces cerevisiae* have already been isolated and characterised from the intestine of different fish species. Their role in the health and nutrition of farmed fish has also been studied in detail, showing a positive effect on the overall health of the host^63^. Members of *Candida* sp. have been reported from the intestinal population of marine fish and a member of the salmonid family rainbow trout (*Oncorhynchus mykiss*)^64,65^, although their specific role in host biology remains to be described.

*Davidiella tassiana* and *Alternaria metachromatica* are known plant pathogens^66^. These two phylotypes occur at different stages of ontogeny, including eyed eggs (EE). Although their functions in teleosts are generally uncharacterised, the susceptibility of fish eggs to fungal infections of multispecies origin may indicate that these groups are indeed opportunistic pathogens. The presence of *Humicola nigrescens* has been detected in environmental soil samples^67^, but its occurrence in the host-associated microbiome is not well established. These groups of organisms are known to produce extracellular phytase that cleaves phytic acid, resulting in higher bioavailability of organic phosphate, zinc and iron^68^. It is likely that this phylotype provides these minerals, which are important for host development, in the early stages of development and in the intestine of young animals and adults.

The fungal microbiota in the early stages of ontogeny and in the intestine of juvenile and adult fish is a mixture of potentially opportunistic pathogenic phylotypes and some fungicidal and probiotic groups. Overall, these results warrant further investigation to understand whether it is indeed an arms race between pathogens and beneficial groups. Furthermore, understanding the role of abiotic factors such as temperature, pH and other water quality parameters in this interaction is crucial for the development of aquaculture health management strategies.

### Fungal diversity is significantly lower than the bacterial diversity in most of the stages of ontogeny

In addition, we compared the alpha diversity of fungi with that of bacteria at the same developmental stages and found that fungal diversity was significantly lower at most stages, with the exception of a higher diversity value in the EBH group. Lower fungal diversity compared to bacterial diversity has been found in many species^65,69^. Although fungal populations have lower diversity compared to bacteria, they are considered functionally important for the stability of the microbial ecosystem because there are specific interactions between bacterial and fungal groups that are important for the physiology of the host^65,70^. Indeed, in zebrafish it has been shown that exposure of larvae to *Debaryomyces* sp. (isolated from the intestine of zebrafish) resulted in a significant increase in the abundance of potentially beneficial bacterial genera such as *Pediococcus* and *Lactococcus*, suggesting possible cross-kingdom interactions between these groups^13^.

### Impact of environment on the composition

The impact of environmental factors on host-associated microbiomes is significant. Experimental manipulations involving both warming and cooling have demonstrated a noteworthy reduction in microbial diversity within various host-associated microbiomes^71^. Notably, studies on chinook salmon (*Oncorhynchus tshawytscha*) have revealed a significant shift in microbial communities when water temperatures are altered, transitioning from a Vibrionaceae-rich community to one dominated by Fusobacteriaceae and Brevinemataceae as temperatures increase from 8 to 20 °C^72^. The influence of salinity on the Atlantic salmon, with a drastic shift from a Firmicute-rich to a proteobacteria-rich community upon transfer to seawater, further underscores the environmental impact on microbiome dynamics^18^. Moreover, the decrease in fungal phylotype diversity following transfer to seawater mirrors similar observations made in bacterial populations undergoing the same environmental transition^55^. In addition to temperature and salinity, factors such as diet and pH have been recognized as influential in teleosts^73,74^. However, in our current study, the fish were consistently fed standard commercial diets with minimal variations, and temperature and pH levels were controlled throughout ontogeny. Consequently, the observed shifts in abundance profiles of fungal phylotypes are likely associated with host development rather than variations in these environmental factors.

Furthermore, while it has been traditionally assumed that the microbe-rich aquatic environment would lead to random colonization of the fish intestine, particularly during early ontogenic stages, recent evidence challenges this notion^75^. Contrary to expectations, the host appears to play a highly selective role in shaping the composition of microbial communities during development^51,76–78^. This selectivity may be tightly regulated by host factors, such as the immune system, which acts as a deterministic force in bacterial group assembly^78^. Future studies should delve into the intricate interactions between host factors and fungal phylotypes to unravel the nuanced dynamics governing host-associated microbiomes.

One limitation of the present study is that the samples in all stages were taken from a single tank and cage (in the case of farms), thereby missing any potential variation arising from the tank effect. Previous studies have demonstrated interactions between the microbiome and reared fish in a tank-specific manner^79–81^. Therefore, this aspect needs to be addressed in future studies. Furthermore, consideration of inherent interindividual variations in the host-associated microbiome is crucial^82^. In this study, alpha diversities in hatchery-reared fish were generally lower than those in farm-sampled fish (Figs. 1A, 2A, 3A, 4A, 5A and 6A). Variations in beta diversity were also relatively lower in embryonic and early intestinal stages (Figs. 1B, 2B). However, as fish developed the variation increased, likely due to exposure to different host and environmental factors (Figs. 3B, 4B, 5B, 6B). Hence for a better overview, it could be recommended to increase the sample size by including individuals from multiple tanks in studies of the host-associated mycobiome in Atlantic salmon.

## Conclusions and future perspectives

**Figure 7:**
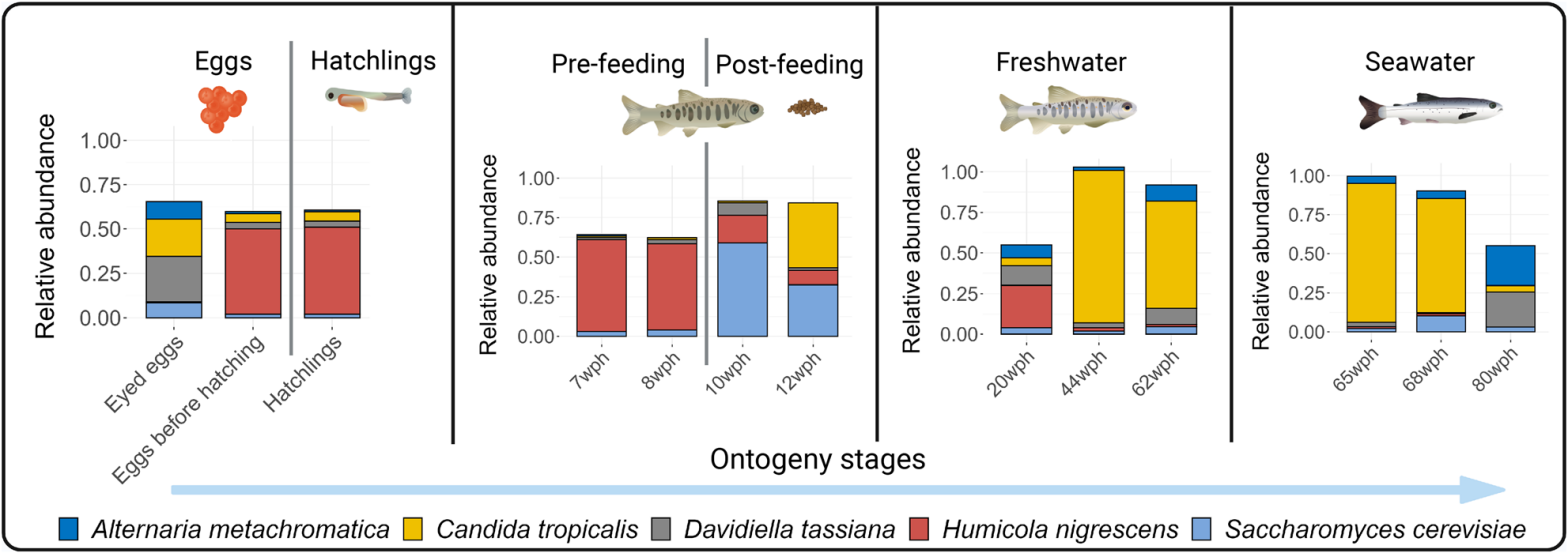
Illustration of the transition in the fungal communities of Atlantic salmon during its life cycle. We show that hatching does not cause a major change in community diversity, whereas the first feeding and the transition to seawater strongly affect the diversity and composition of the fungal population in the intestine. Some of the major phylotypes, including *Humicola nigrescens*, *Saccharomyces cerevisiae*, *Candida tropicalis*, *Davidiella tassiana* and *Alternaria metachromatica*, were present at all stages of development, and their abundance varied at different stages of ontogeny.

In the present study, we report the transition in fungal communities in early life stages and intestine of juvenile and adult Atlantic salmon. Fungal alpha diversity varies across developmental stages, as has been observed for bacterial alpha diversity. Such variation in fungal diversity has been observed previously in humans, and some of the changes have been attributed to factors such as nutrition and immune system development. The overall diversity of fungi was lower compared to bacteria and there were significant changes in abundance profiles during developmental stages. Core groups of phylotypes that were persistently present in all developmental stages and fish from different farms included *Humicola nigrescens*, *Saccharomyces cerevisiae*, *Candida tropicalis*, *Davidiella tassiana*, and *Alternaria metachromatica* (Fig. 7). These phylotypes include potential opportunistic pathogens as well as fungicidal strains used as probiotics. Key events such as the first feeding and transfer to seawater had a strong influence on the composition of the fungal community and led to a shift in the abundance profile. Spatial differences in the diversity of fungal communities from different farms were minimal, although there was a farm-specific abundance profile. The current study shows that fungal communities in Atlantic salmon are highly diverse and may represent an important area of research pertaining to the host microbiome interactions.

### Supplementary Information

Supplementary Information.

## Data Availability

All sequence data are available at the NCBI sequence read archive under accession number PRJNA919076. https://dataview.ncbi.nlm.nih.gov/object/PRJNA919076?reviewer=ra7v0vg28nen0l9fg3ncg1lf6a.

## References

[1] Berg G (2020). Microbiome definition re-visited: Old concepts and new challenges. Microbiome.

[2] Frey-Klett P (2011). Bacterial-fungal interactions: hyphens between agricultural, clinical, environmental, and food microbiologists. Microbiol. Mol. Biol. Rev. MMBR.

[3] Gu Y (2019). The potential role of gut mycobiome in irritable bowel syndrome. Front. Microbiol..

[4] Hagen LH (2021). Proteome specialization of anaerobic fungi during ruminal degradation of recalcitrant plant fiber. ISME J..

[5] Underhill DM, Iliev ID (2014). The mycobiota: Interactions between commensal fungi and the host immune system. Nat. Rev. Immunol..

[6] Nash AK (2017). The gut mycobiome of the human microbiome project healthy cohort. Microbiome.

[7] David LA (2014). Diet rapidly and reproducibly alters the human gut microbiome. Nature.

[8] Kumar S, Indugu N, Vecchiarelli B, Pitta DW (2015). Associative patterns among anaerobic fungi, methanogenic archaea, and bacterial communities in response to changes in diet and age in the rumen of dairy cows. Front. Microbiol..

[9] Schei K (2017). Early gut mycobiota and mother-offspring transfer. Microbiome.

[10] Jiang H (2020). Impact of host intraspecies genetic variation, diet, and age on bacterial and fungal intestinal microbiota in tigers. MicrobiologyOpen.

[11] Siriyappagouder P (2018). The intestinal mycobiota in wild zebrafish comprises mainly *Dothideomycetes* while *Saccharomycetes* predominate in their laboratory-reared counterparts. Front. Microbiol..

[12] Siriyappagouder P (2020). *Pseudozyma* priming influences expression of genes involved in metabolic pathways and immunity in zebrafish larvae. Front. Immunol..

[13] Siriyappagouder P (2018). Exposure to yeast shapes the intestinal bacterial community assembly in zebrafish larvae. Front. Microbiol..

[14] Zhou L (2021). Comparison of fungal community composition within different intestinal segments of tilapia and bighead carp. J. Oceanol. Limnol..

[15] Marden CL (2017). Investigation into the fungal diversity within different regions of the gastrointestinal tract of *Panaque nigrolineatus*, a wood-eating fish. AIMS Microbiol..

[16] Reinoso S (2023). Feed regime slightly modifies the bacterial but not the fungal communities in the intestinal mucosal microbiota of cobia fish (*Rachycentron canadum*). Microorganisms.

[17] Raggi P (2014). *Debaryomyces hansenii* and *Rhodotorula mucilaginosa* comprised the yeast core gut microbiota of wild and reared carnivorous salmonids, croaker and yellowtail. Environ. Microbiol..

[18] Lokesh, J., Kiron, V., Sipkema, D., Fernandes, J. M. O. & Moum, T. Succession of embryonic and the intestinal bacterial communities of Atlantic salmon (*Salmo salar*) reveals stage-specific microbial signatures. *MicrobiologyOpen* **8**, (2019). 10.1002/mbo3.672 PMC6460355 29897674

[19] NRC. *Nutrient Requirements of Fish*. (National Academies Press, 1993). 10.17226/2115.

[20] Lokesh J, Kiron V (2016). Transition from freshwater to seawater reshapes the skin-associated microbiota of Atlantic salmon. Sci. Rep..

[21] Kozich JJ, Westcott SL, Baxter NT, Highlander SK, Schloss PD (2013). Development of a dual-index sequencing strategy and curation pipeline for analyzing amplicon sequence data on the MiSeq Illumina sequencing platform. Appl. Environ. Microbiol..

[22] Ihrmark K (2012). New primers to amplify the fungal ITS2 region—evaluation by 454-sequencing of artificial and natural communities. FEMS Microbiol. Ecol..

[23] Edgar RC (2013). UPARSE: Highly accurate OTU sequences from microbial amplicon reads. Nat. Methods.

[24] Gweon HS (2015). PIPITS: An automated pipeline for analyses of fungal internal transcribed spacer sequences from the Illumina sequencing platform. Methods Ecol. Evol..

[25] Wang Q, Garrity GM, Tiedje JM, Cole JR (2007). Naive bayesian classifier for rapid assignment of rRNA sequences into the new bacterial taxonomy. Appl. Environ. Microbiol..

[26] Kõljalg U (2005). UNITE: A database providing web-based methods for the molecular identification of ectomycorrhizal fungi. New Phytol..

[27] R Core Team. R: A language and environment for statistical computing. (2018).

[28] McMurdie PJ, Holmes S (2013). phyloseq: An R package for reproducible interactive analysis and graphics of microbiome census data. PLoS ONE.

[29] Clarke KR, Warwick RM (1994). Similarity-based testing for community pattern: The two-way layout with no replication. Mar. Biol..

[30] Oksanen, J. *et al.* vegan: Community ecology package. (2018).

[31] Caporaso JG (2010). QIIME allows analysis of high-throughput community sequencing data. Nat. Methods.

[32] Segata N (2011). Metagenomic biomarker discovery and explanation. Genome Biol..

[33] Liu Y (2014). Deciphering microbial landscapes of fish eggs to mitigate emerging diseases. ISME J..

[34] Thambugala, K. M., Daranagama, D. A., Phillips, A. J. L., Kannangara, S. D. & Promputtha, I. Fungi vs. Fungi in biocontrol: An overview of fungal antagonists applied against fungal plant pathogens. *Front. Cell. Infect. Microbiol.* **10**, (2020). 10.3389/fcimb.2020.604923 PMC7734056 33330142

[35] Řehulka J, Kubátová A, Hubka V (2016). *Cephalotheca sulfurea* (Ascomycota, Sordariomycetes), a new fungal pathogen of the farmed rainbow trout *Oncorhynchus mykiss*. J. Fish Dis..

[36] Nenoff, P., Krüger, C., Ginter-Hanselmayer, G. & Tietz, H.-J. Mycology—an update. Part 1: Dermatomycoses: causative agents, epidemiology and pathogenesis. *JDDG J. Dtsch. Dermatol. Ges.* **12**, 188–210 (2014). 10.1111/ddg.12245 24533779

[37] Ali SS (2019). Draft genome sequence of fastidious pathogen *Ceratobasidium theobromae*, which causes vascular-streak dieback in *Theobroma cacao*. Fungal Biol. Biotechnol..

[38] de Hoog GS (2013). Phylogenetic findings suggest possible new habitat and routes of infection of human Eumyctoma. PLoS Negl. Trop. Dis..

[39] Clark ES, Wilkins LGE, Wedekind C (2013). MHC class I expression dependent on bacterial infection and parental factors in whitefish embryos (Salmonidae). Mol. Ecol..

[40] Wilkins, L. G. E., Rogivue, A., Fumagalli, L. & Wedekind, C. Declining diversity of egg-associated bacteria during development of naturally spawned whitefish embryos (Coregonus spp.). *Aquat. Sci.* **77**, 481–497 (2015).

[41] Wilkins LGE, Rogivue A, Schütz F, Fumagalli L, Wedekind C (2015). Increased diversity of egg-associated bacteria on brown trout (*Salmo trutta*) at elevated temperatures. Sci. Rep..

[42] Bledsoe JW, Pietrak MR, Burr GS, Peterson BC, Small BC (2022). Functional feeds marginally alter immune expression and microbiota of Atlantic salmon (*Salmo salar*) gut, gill, and skin mucosa though evidence of tissue-specific signatures and host-microbe coadaptation remain. Anim. Microbiome.

[43] Llewellyn MS, Boutin S, Hoseinifar SH, Derome N (2014). Teleost microbiomes: the state of the art in their characterization, manipulation and importance in aquaculture and fisheries. Front. Microbiol..

[44] Phelps D (2017). Microbial colonization is required for normal neurobehavioral development in zebrafish. Sci. Rep..

[45] Kanther M, Rawls JF (2010). Host–microbe interactions in the developing zebrafish. Curr. Opin. Immunol..

[46] Murdoch, C. C. & Rawls, J. F. Commensal microbiota regulate vertebrate innate immunity: Insights from the zebrafish. *Front. Immunol.* **10**, (2019). 10.3389/fimmu.2019.02100 PMC6742977 31555292

[47] Rawls JF, Samuel BS, Gordon JI (2004). Gnotobiotic zebrafish reveal evolutionarily conserved responses to the gut microbiota. Proc. Natl. Acad. Sci..

[48] Houhamdi Hammou, L. *et al. Malassezia restricta*: An underdiagnosed causative agent of blood culture-negative infective endocarditis. *Clin. Infect. Dis. Off. Publ. Infect. Dis. Soc. Am.* **73**, 1223–1230 (2021). 10.1093/cid/ciab377 34009270

[49] Pradhan PC (2022). Performance appraisal of *Trichoderma viride* based novel tablet and powder formulations for management of Fusarium wilt disease in chickpea. Front. Plant Sci..

[50] Hamre K (2013). Fish larval nutrition and feed formulation: knowledge gaps and bottlenecks for advances in larval rearing. Rev. Aquac..

[51] Bakke I, Coward E, Andersen T, Vadstein O (2015). Selection in the host structures the microbiota associated with developing cod larvae (*Gadus morhua*). Environ. Microbiol..

[52] Zhang, Z., Li, D., Xu, W., Tang, R. & Li, L. Microbiome of co-cultured fish exhibits host selection and niche differentiation at the organ scale. *Front. Microbiol.* **10**, (2019). 10.3389/fmicb.2019.02576 PMC6856212 31781072

[53] de Barros, P. P. *et al. Candida tropicalis* affects the virulence profile of *Candida albicans*: an in vitro and in vivo study. *Pathog. Dis.* **76**, (2018). 10.1093/femspd/fty014 29617858

[54] McCormick SD, Hansen LP, Quinn TP, Saunders RL (1998). Movement, migration, and smolting of Atlantic salmon (*Salmo salar*). Can. J. Fish. Aquat. Sci..

[55] Dehler, C. E., Secombes, C. J. & Martin, S. A. M. Seawater transfer alters the intestinal microbiota profiles of Atlantic salmon (*Salmo salar* L.). *Sci. Rep.* **7**, 13877–13877 (2017). 10.1038/s41598-017-13249-8 PMC5654775 29066818

[56] Kim PS (2021). Host habitat is the major determinant of the gut microbiome of fish. Microbiome.

[57] Lorgen-Ritchie M (2023). Time is a stronger predictor of microbiome community composition than tissue in external mucosal surfaces of Atlantic salmon (*Salmo salar*) reared in a semi-natural freshwater environment. Aquaculture.

[58] Giatsis C (2015). The impact of rearing environment on the development of gut microbiota in tilapia larvae. Sci. Rep..

[59] Sullam KE (2012). Environmental and ecological factors that shape the gut bacterial communities of fish: A meta-analysis. Mol. Ecol..

[60] Luan Y (2023). The fish microbiota: Research progress and potential applications. Engineering.

[61] Alconada, T. M. & Martínez, M. J. Purification and characterization of a β‐glucosidase from the phytopathogenic fungus *Fusarium oxysporum. sp. melonis*. *Lett. Appl. Microbiol.* **22**, 106–110 (1996).

[62] Sharp C, Foster KR (2022). Host control and the evolution of cooperation in host microbiomes. Nat. Commun..

[63] Sharma S, Dahiya T, Jangra M, Muwal A, Singh C (2022). *Saccharomyces cerevisiae* as probiotics in aquaculture. J. Entomol. Zool. Stud..

[64] Andlid T, Juárez RV, Gustafsson L (1995). Yeast colonizing the intestine of rainbow trout (*Salmo gairdneri*) and turbot (*Scophtalmus maximus*). Microb. Ecol..

[65] Gatesoupe FJ (2007). Live yeasts in the gut: Natural occurrence, dietary introduction, and their effects on fish health and development. Aquaculture.

[66] Bashir U, Mushtaq S, Akhtar N (2014). First report of *Alternaria metachromatica* from Pakistan causing leaf spot of tomato. Pak. J. Agric. Sci..

[67] Sharma-Poudyal D, Schlatter D, Yin C, Hulbert S, Paulitz T (2017). Long-term no-till: A major driver of fungal communities in dryland wheat cropping systems. PloS One.

[68] Bala, A., Sapna, Jain, J., Kumari, A. & Singh, B. Production of an extracellular phytase from a thermophilic mould *Humicola nigrescens* in solid state fermentation and its application in dephytinization. *Biocatal. Agric. Biotechnol.* **3**, 259–264 (2014).

[69] Scanlan PD, Marchesi JR (2008). Micro-eukaryotic diversity of the human distal gut microbiota: qualitative assessment using culture-dependent and -independent analysis of faeces. ISME J..

[70] Lapiere A, Richard ML (2022). Bacterial-fungal metabolic interactions within the microbiota and their potential relevance in human health and disease: A short review. Gut Microbes.

[71] Li J (2023). Experimental temperatures shape host microbiome diversity and composition. Glob. Change Biol..

[72] Steiner K, Laroche O, Walker SP, Symonds JE (2022). Effects of water temperature on the gut microbiome and physiology of Chinook salmon (*Oncorhynchus tshawytscha*) reared in a freshwater recirculating system. Aquaculture.

[73] Li Y (2021). Differential response of digesta- and mucosa-associated intestinal microbiota to dietary insect meal during the seawater phase of Atlantic salmon. Anim. Microbiome.

[74] Sylvain F-É (2016). pH drop impacts differentially skin and gut microbiota of the Amazonian fish tambaqui (*Colossoma macropomum*). Sci. Rep..

[75] Burns AR (2016). Contribution of neutral processes to the assembly of gut microbial communities in the zebrafish over host development. ISME J..

[76] Yan Q (2016). Environmental filtering decreases with fish development for the assembly of gut microbiota. Environ. Microbiol..

[77] Pratte ZA, Besson M, Hollman RD, Stewart FJ (2018). The gills of reef fish support a distinct microbiome influenced by host-specific factors. Appl. Environ. Microbiol..

[78] Stagaman K, Burns AR, Guillemin K, Bohannan BJ (2017). The role of adaptive immunity as an ecological filter on the gut microbiota in zebrafish. ISME J..

[79] Minich JJ (2020). Microbial ecology of Atlantic salmon (*Salmo salar*) hatcheries: Impacts of the built environment on fish mucosal microbiota. Appl. Environ. Microbiol..

[80] Uren Webster, T. M., Rodriguez-Barreto, D., Consuegra, S. & Garcia de Leaniz, C. Cortisol-related signatures of stress in the fish microbiome. *Front. Microbiol.* **11**, (2020). 10.3389/fmicb.2020.01621 PMC7381252 32765459

[81] Bone A (2021). Bacterial communities of Ballan Wrasse (*Labrus bergylta*) eggs at a commercial marine hatchery. Curr. Microbiol..

[82] Healey GR, Murphy R, Brough L, Butts CA, Coad J (2017). Interindividual variability in gut microbiota and host response to dietary interventions. Nutr. Rev..

