# Supplementary material for: Unravelling the temporal and spatial variation of fungal phylotypes from embryo to adult stages in Atlantic salmon
Source: Sci Rep. 2024 Jan 10;14:981. doi: 10.1038/s41598-023-50883-x (PMC10781754; doi:10.1038/s41598-023-50883-x)
Supplement: Supplementary file 1 — Supplementary Information. [file 41598_2023_50883_MOESM1_ESM.pdf]

Supplementary materials

Unravelling the temporal and spatial variation of fungal phylotypes from embryo to adult stages in Atlantic salmon

Jep Lokesh[1]#*, Prabhugouda Siriyappagouder[1], Jorge M.O. Fernandes[1]*

1Faculty of Biosciences and Aquaculture, Nord University, Bodø, Norway

#Current address: Université de Pau et des Pays de l'Adour, E2S UPPA. INRAE, NUMEA, Saint-Pée-sur-Nivelle, France

Running title: Fungal communities of Atlantic salmon

Keywords: Aquaculture, Atlantic salmon (Salmo salar), fungal community, microbiota, intestine, amplicon sequencing.

* Corresponding authors: lokesh.jep@inrae.fr; jorge.m.fernandes@nord.no

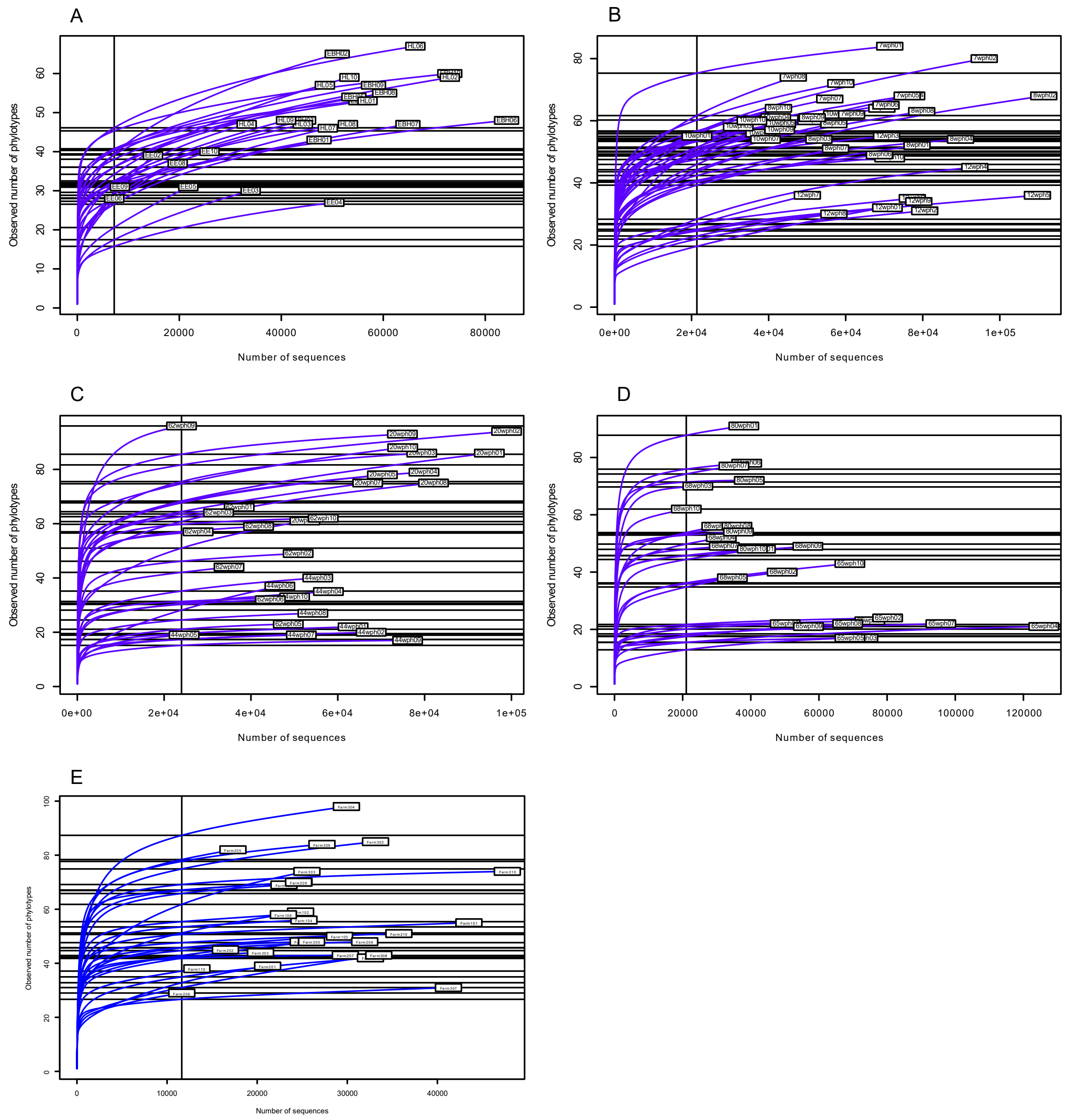

Supplementary Figure 1: Rarefaction curves for different samples during (A) the early stages of ontogeny (EE, EBH, and HL), (B) the intestine of early stages (7 wph, 8 wph, 10 wph, and 12 wph), (C) the intestine of the late freshwater stages (20 wph, 44 wph, and 62 wph), (D) the seawater phase (65 wph, 68 wph, and 80 wph), and (E) three different farms.

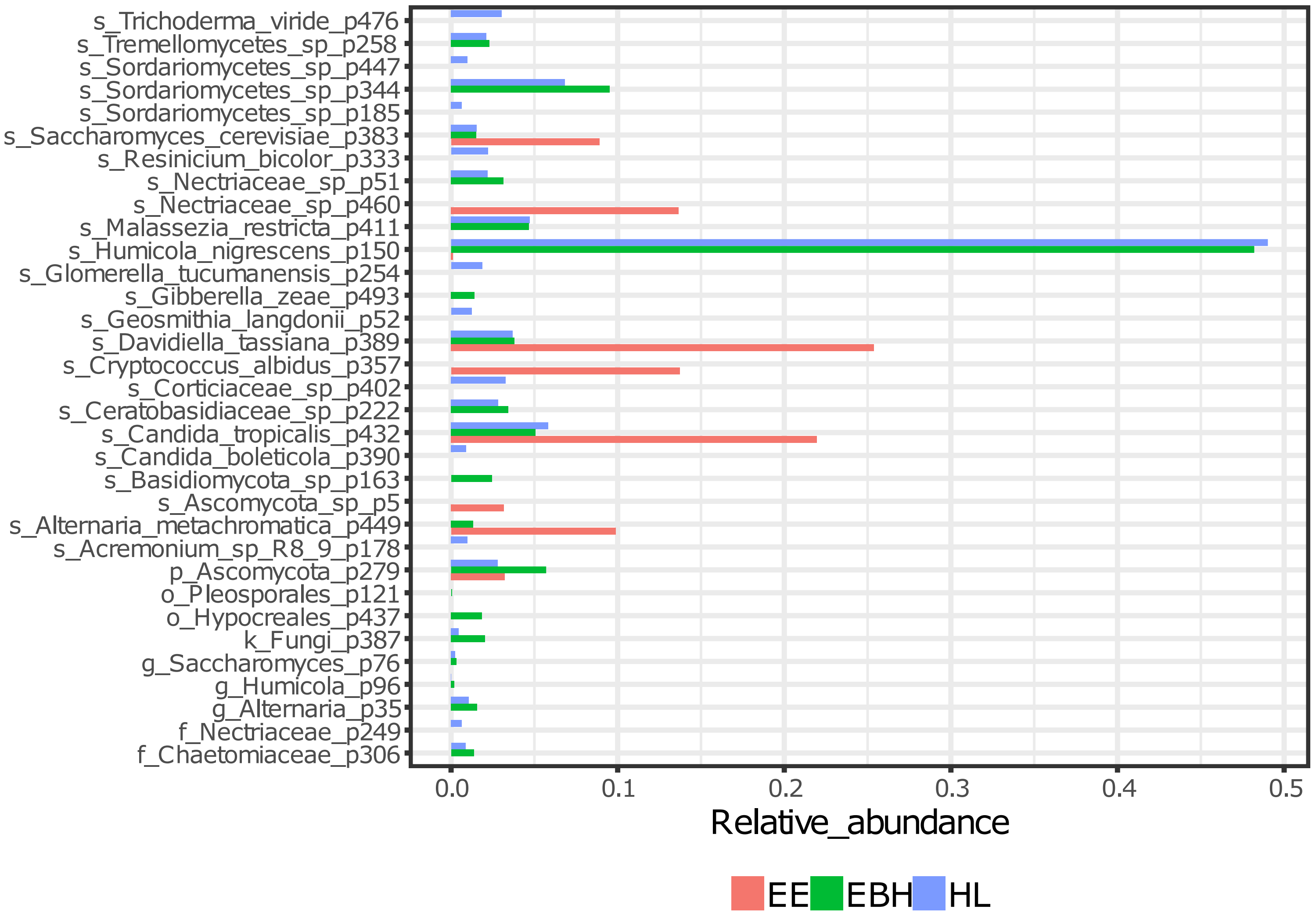

Supplementary figure 2: Composition of the core phylotypes during the early stages of ontogeny (EE, EBH, and HL) with a minimum relative abundance greater than 0.01.

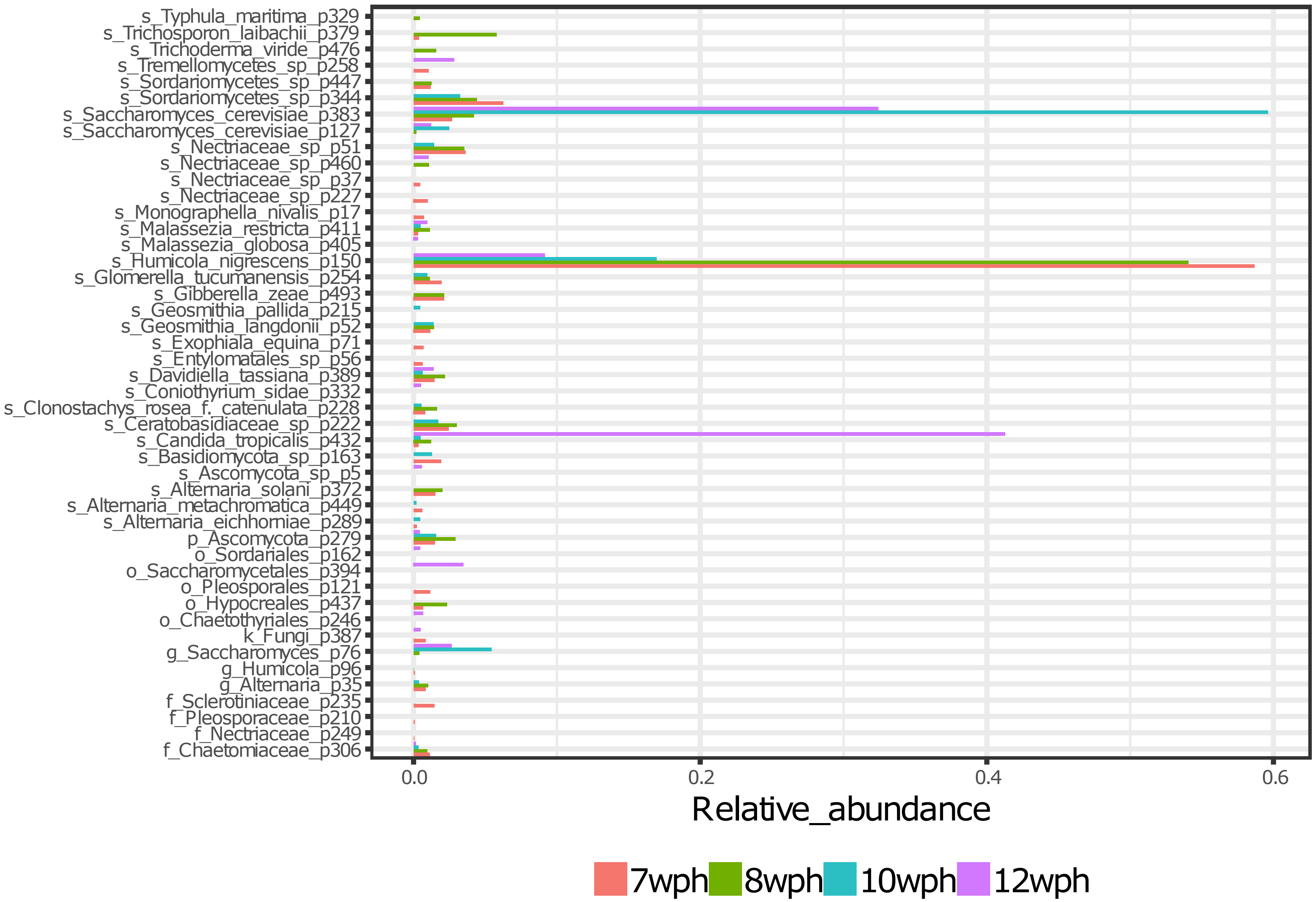

Supplementary figure 3: Composition of the core phylotypes in the intestine of the early stages of ontogeny (7 wph, 8 wph, 10 wph and 12 wph) with a minimum relative abundance greater than 0.01.

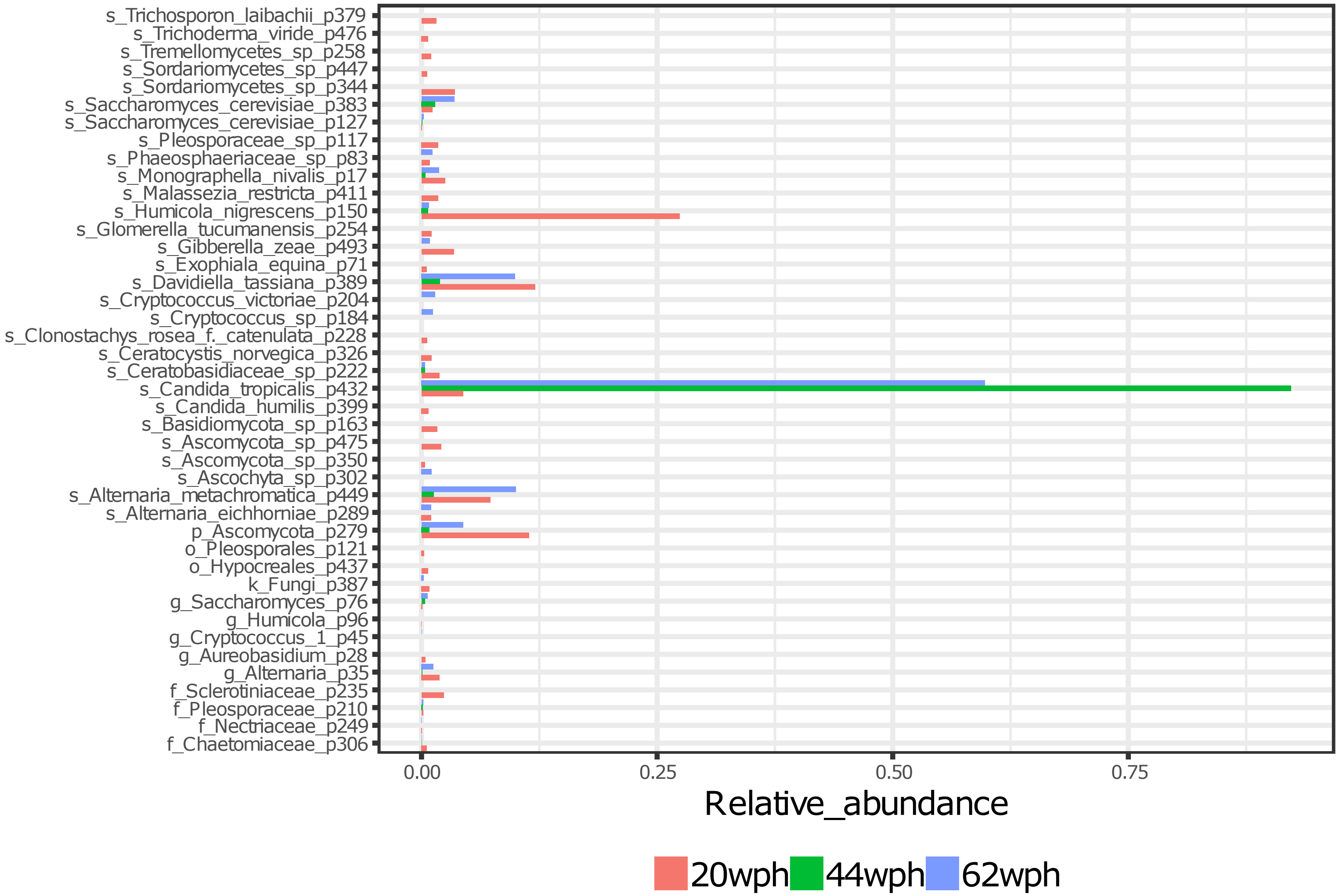

Supplementary figure 4: Composition of the core phylotypes in the intestine of the late freshwater stages of Atlantic salmon (20 wph, 44 wph, and 62 wph) with a minimum relative abundance greater than 0.01.

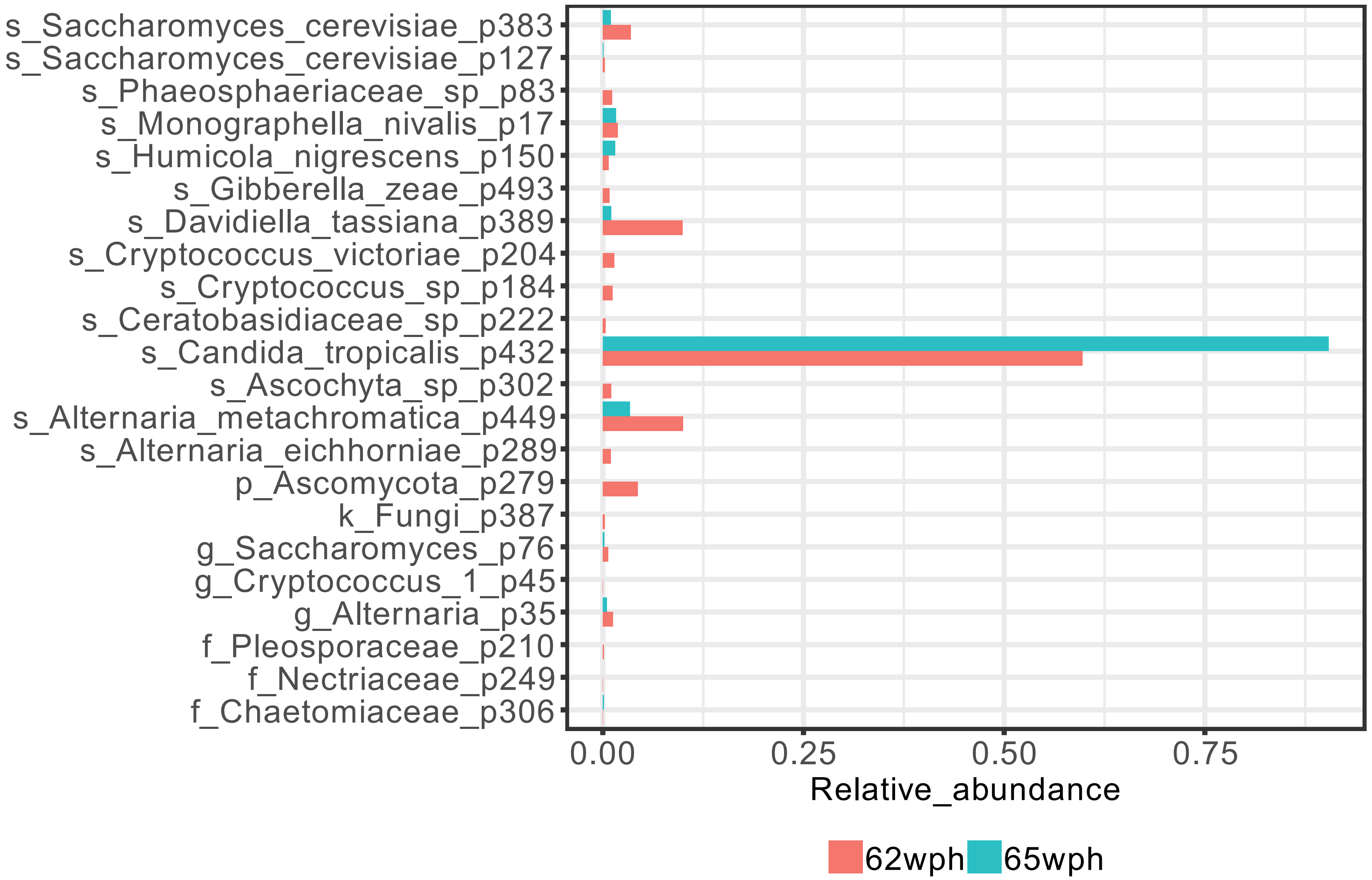

Supplementary figure 5: Composition of the core phylotypes (minimum relative abundance greater than 0.01) in the intestine of Atlantic salmon during the transition from freshwater (62 wph) to seawater (65 wph).

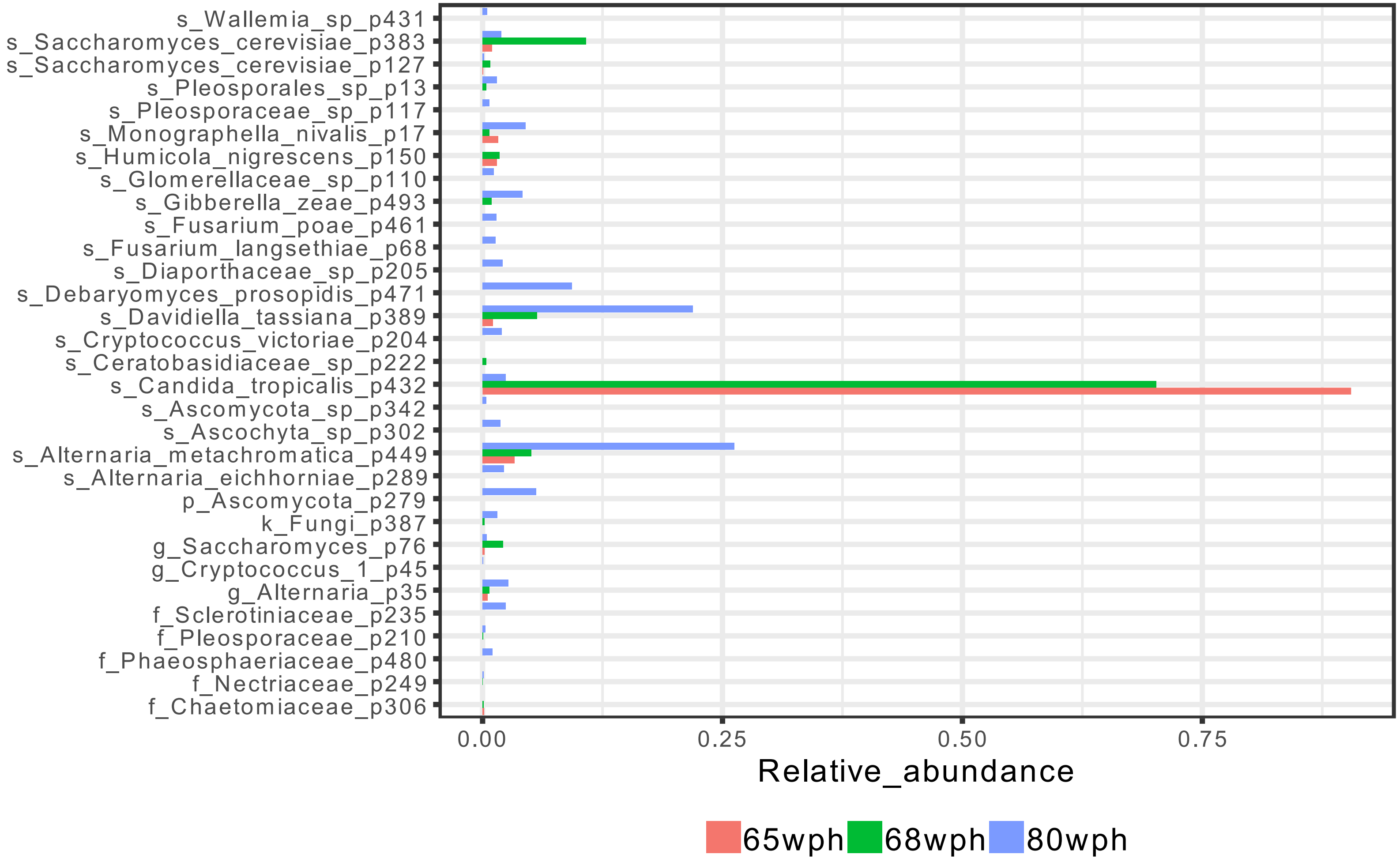

Supplementary figure 6: Composition of the core phylotypes during the stages of ontogeny sampled from the seawater phase (65 wph, 68 wph, and 80 wph) with a minimum relative abundance greater than 0.01.

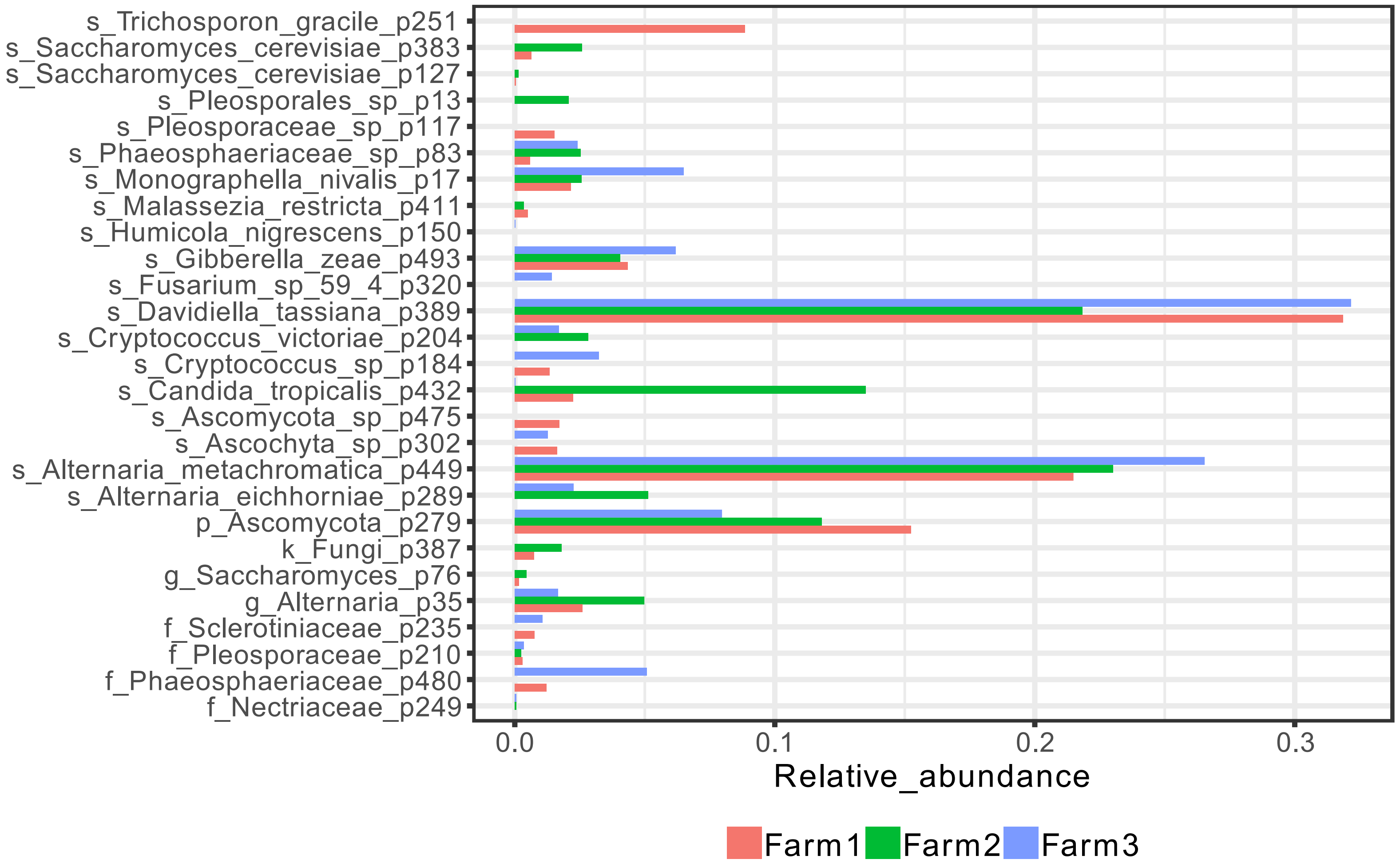

Supplementary figure 7: Composition of the core phylotypes of the Atlantic salmon originating from three different with a minimum relative abundance greater than 0.01.

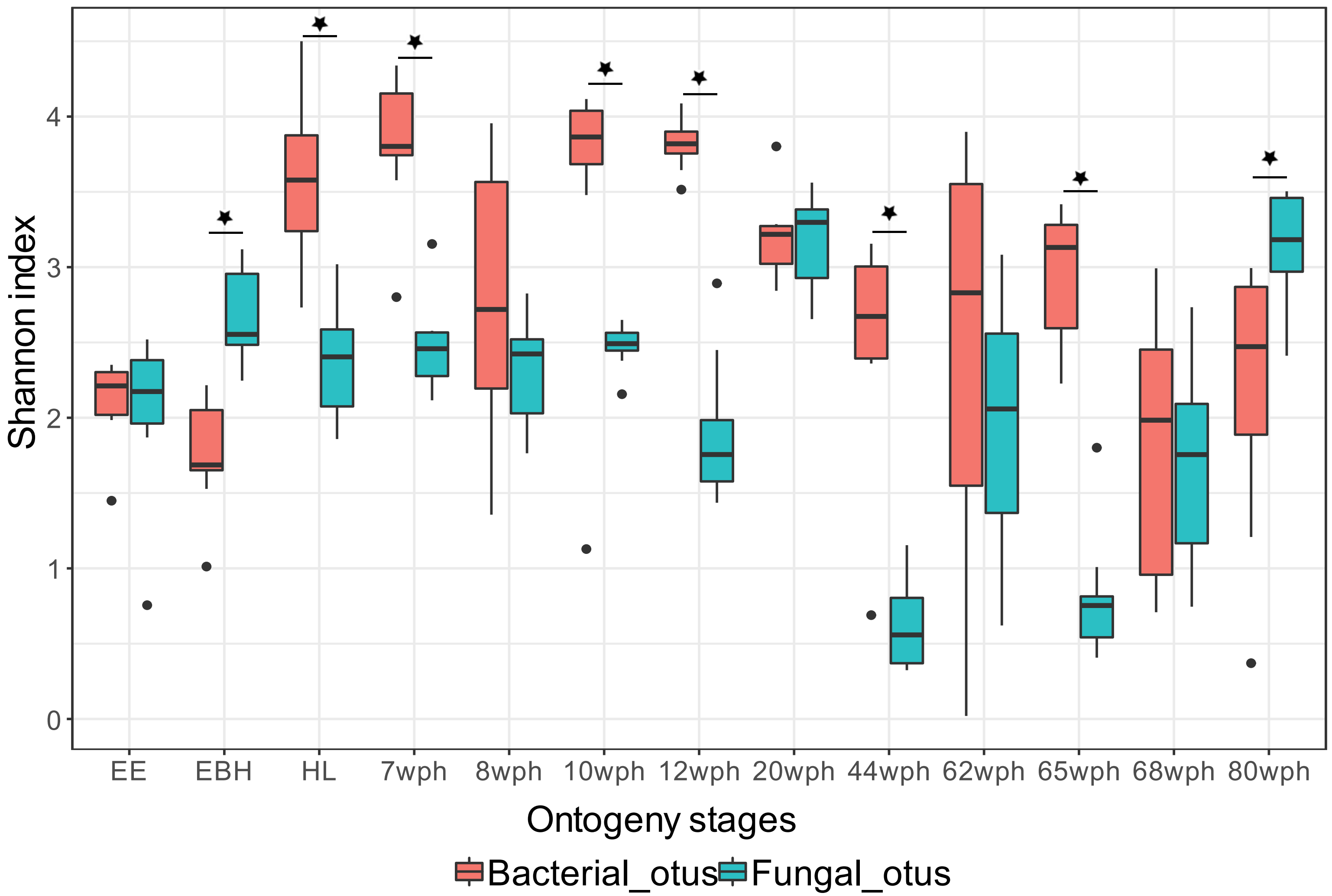

Supplementary figure 8: Comparison of the bacterial and fungal alpha diversities at different stages of ontogeny of Atlantic salmon.

Supplementary table 1: Nutrient composition of the diets used in different stage of development and farms in the present study.

Nutrient composition (% dry feed basis)	Starter feed	Smolt feed	Grower feed
Crude protein	50	45	45
Crude lipid	18	20	25
Carbohydrate	10	12	12
Crude fibre	2	3	3
Digestible energy (kJ/g)	19	19	20
